# A Novel Image-Guided, Automatic, High-Intensity Neurostimulation Device for the Treatment of Nonspecific Low Back Pain

**DOI:** 10.1155/2011/152307

**Published:** 2011-04-21

**Authors:** Miguel Gorenberg, Elad Schiff, Kobi Schwartz, Elon Eizenberg

**Affiliations:** ^1^Department of Nuclear Medicine, Bnai Zion Medical Center, Haifa, Israel; ^2^The Rappaport Faculty of Medicine, Technion—Israel Institute of Technology, Haifa 32000, Israel; ^3^Department of Internal Medicine, Bnai Zion Medical Center, Haifa, Israel; ^4^Department of Physical Therapy, Bnai Zion Medical Center, Haifa, Israel; ^5^Pain Relief Unit, Rambam Medical Center, Haifa 31096, Israel

## Abstract

*Purpose*. The current pilot study investigates the effectiveness of a novel device in the management of chronic low back pain (LBP). This device is able to automatically measure skin impedance in a selected body area and, immediately afterwards, to stimulate multiple points that are targeted according to differentiation in their electrical properties (peripheral nerve ends—milinated A *δ* fibers) with high-intensity electrical stimulation. *Materials and Methods*. Nineteen outpatients were included in the study, 15 females (79%) and 4 men (21%), mean age 52.1 ± 10.8 years, all diagnosed with nonspecific chronic LBP. The protocol consisted of 6 treatment sessions, 2–4 days apart. Each session included a <1 minute automatic impedance screening, followed by a 20-minute treatment of lowest impedance points according to proprietary algorithms. *Outcome Measures*. The primary outcome measure consisted of changes in pain intensity as measured on a 100 mm pain visual analogue scale (VAS) obtained at enrollment, before and 2 hours after each treatment. Secondary outcome measures were the Oswestry Disability Index (ODI) and lumbar flexion range of motion (ROM) obtained at baseline and each week during treatment. *Results*. The mean ± SD baseline VAS score for all participants was 61 ± 14. There were no significant changes in VAS scores between enrollment and before the first treatment (55 ± 16; *P* = .102). During treatment, VAS scores decreased significantly compared with baseline scores by 39 ± 17 mm (*P* < .001). Notably, VAS scores of all the patients, except for one, decreased by more than 20 mm after the fourth treatment, thus showing marked improvement in 95% of enrolled patients. ODI decreased throughout the entire treatment period, with significant changes from baseline already at the first week (*P* = .001). Lumbar flexion ROM showed a mean increase of 2.1 cm during treatment, but was not statistically significant. *Conclusion*. The results of the current pilot study show that treatment with this novel device produced a clinically significant reduction in back pain in 95% of patients after four treatment sessions. The decrease both in pain and perceived disability, combined with the improvement in ROM, support further investigation of the use of this therapy in the treatment of LBP.

## 1. Introduction

Despite the fact that low back pain (LBP) is one of the most common medical problems in our society [1], current analgesic therapies remain largely unsatisfactory. Conservative treatment with anti-inflammatory drugs and exercise is effective for many patients with acute LBP [2]. However, when the pain symptoms persist, they can interfere with both physical activity and sleep patterns. While analgesic medications can provide temporary pain relief, these drugs may not improve physical function and are associated with well-known adverse effects [3]. These concerns have increased interest in nonpharmacological therapies for LBP, such as transcutaneous electrical nerve stimulation (TENS) [4], electroacupuncture [5], and percutaneous electrical nerve stimulation (PENS) [6–8]. Unfortunately, these alternatives fall short with respect to duration and magnitude of analgesia.

Localized manual high-intensity neurostimulation devices are applied to small surface areas to stimulate peripheral nerve endings (A *δ* fibers), thus causing the release of endogenous endorphins [9, 10]. This procedure, often described as “hyperstimulation analgesia”, has been investigated in several controlled studies, showing a positive response in 87% of patients [9]. However, such treatments are time consuming and cumbersome, and require previous knowledge of the localization of peripheral nerve endings responsible for LBP or manual impedance mapping of the back. These limitations prevent their extensive utilization [9, 11]. 

The purpose of the present pilot study was to assess the effectiveness of a novel device capable of automatically measuring skin impedance in a selected body area and, immediately afterwards, of stimulating multiple points that are targeted according to differentiation in their electrical properties and proprietary image processing algorithms with high intensity yet nonpainful electrical stimulation (Nervo-Stim, Nervomatrix Ltd., Netanya, Israel).

## 2. Methods

### 2.1. Selection of Participants

This pilot study was conducted at the Bnai Zion Medical Center, Department of Physiotherapy from August 2009 to February 2010. Subjects with chronic nonspecific LBP were recruited through the hospital's e-mail system, and most of the participants belonged to the staff working at the hospital. Participants were screened using an LBP examination form and participant history sheet. Their recruitment to the study was based on strict inclusion and exclusion criteria (Table 1). Nonspecific LBP was defined as pain below the twelfth costal margin and above the inferior gluteal folds, not attributed to recognizable, known, specific pathology (e.g., infection, tumour, osteoporosis, ankylosing spondylitis, fracture, inflammatory process, radicular syndrome, or cauda equine syndrome). Screening of all participants was carried out by a qualified physiotherapist who determined, by subjective and objective examination, whether the participants had nonspecific LBP. Ethics committee approval was obtained for this study, and all participants gave informed consent. NIH clinical trial 01132300.

## 3. Study Design

**Figure 1:**
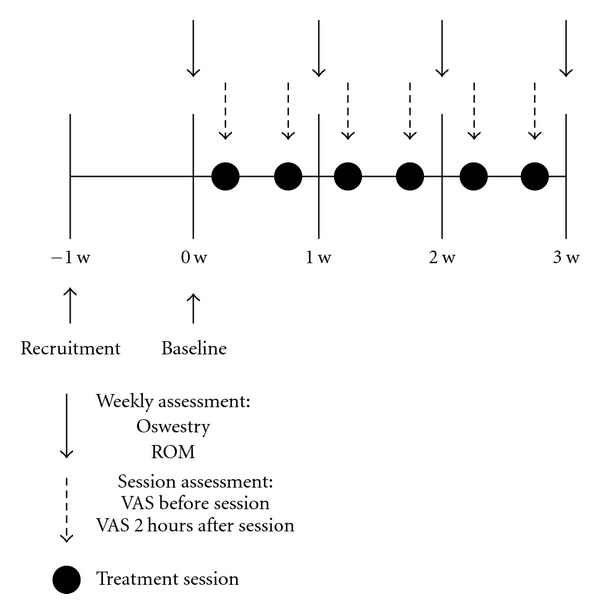
Treatment protocol, which consisted of 6 sessions, twice-weekly, for 3 weeks. Participants completed VAS measurements at enrollment, before and 2 hours after each treatment. Measurements ROM of the lower back and completion of ODI were obtained at enrollment and each week during the study.

All subjects followed the treatment protocol, which consisted of 6 sessions, twice-weekly, for 3 weeks (Figure 1). Each session included an automatic screening (<1 minute) and treatment for 20 minutes with the Nervo-Stim device. 

Participants completed VAS measurements at enrollment, before and 2 hours after each treatment. Measurements for range of motion (ROM) of the lower back and completion of the Oswestry Disability Index (ODI) were obtained at enrollment and each week during the study. The examiners, licensed physical therapists (HV, NB), applied the devices and obtained all VAS, ODI, and ROM measurements. Subjects were advised to avoid new treatments during the time of the study and were instructed not to alter their usual pattern of medication use. 

After enrollment, the subjects underwent a 1-week preobservation period, after which treatment was initiated. The sessions were conducted between 9 AM and 3 PM. Ten of the identified points by the Nervo-Stim within the screened area of LBP were automatically selected for treatment according to proprietary algorithms based on their lowest impedance measurements. Electrical stimulation was performed on these 10 selected points for 20 minutes (each point for 2 minutes, in a consecutive order). 

The electrical current was DC, and the duty cycle was continuous. Lowest impedance points were stimulated at 4/30 Hz. The intensity of the electrical stimulation was adjusted to produce the most intense yet not painful tolerable electrical sensation.

### 3.1. Sample Size

This study was considered a preliminary study in preparation for a main randomized control trial (RCT); therefore, a sample of 20 participants was considered appropriate.

### 3.2. Technology Description

A device, designed to automatically locate peripheral nerve endings relevant for LBP and to deliver high-intensity electrical pulses to these areas, has been developed. Using an array of miniature probes, this automated, computer-controlled device is capable of instant impedance scanning of the entire lower back (max scanned area size 20 × 30 cm^2^) in less than 1 minute. It analyzes the 2-dimensional data based on the measured electrical properties of each finite pixel within the scanned region by using image processing software and algorithms and delivers high-intensity electrical pulses at selected locations. The square wave electrical pulses' frequency range is 1–8 Hz, pulse width is 50–800 *μ*S with a current of 0.1–40 mA.

### 3.3. Outcome Measures

A series of valid and reliable outcome measures were used to assess the different aspects of factors related to LBP. 

The visual analog scale (VAS) was chosen as the primary outcome measure and used to quantify pain intensity. The VAS, shown to be a reliable and valid measure, consists of a standard 100 mm line with verbal anchors indicating “none” at one end (0) and “worst imaginable pain” at the other (100). Participants were told to estimate their current level of pain by indicating an appropriate mark on the line. A 20 mm reduction in pain intensity, measured on the VAS scale, resulting from the treatment, was defined as minimal clinically significant intensity differences (MCID) [12–14]. Secondary outcome measures were the Oswestry Disability Index (ODI) and lumbar flexion range of motion (ROM). The Oswestry Disability Index is a self-completed questionnaire by the patient which examines perceived levels of disability in 10 everyday activities of daily living to assign a subjective score of level of function. Minimal detectable change (90% confidence) is 10% while a smaller change may be attributable to error in the measurement [15, 16]. 

Flexion Hip-Lumbar Spine Range of Motion (ROM): finger-to-floor method involved the subject standing with feet on either side while a measurement of distance in centimeters above and below the knee obtained after each week of treatment was compared to baseline values. Patients that demonstrated a full range of motion at baseline (finger reaching the floor) were not included in the analysis.

### 3.4. Statistical Analysis

Categorical data are presented as absolute and relative frequencies. 

Continuous data are presented as means ± SD, median, and range. Comparisons between various stages in the treatment series were analyzed with paired sample *t*-tests. Whenever a statistically significant difference was found, a 95% confidence interval of the difference was calculated. Differences between the weeks were analyzed with the Wilcoxon Signed Ranks Test. The critical level of significance was 0.05 and the *P* values presented are always 2 sided. The statistical software used was SPSS 17.0.

## 4. Results

**Figure 2:**
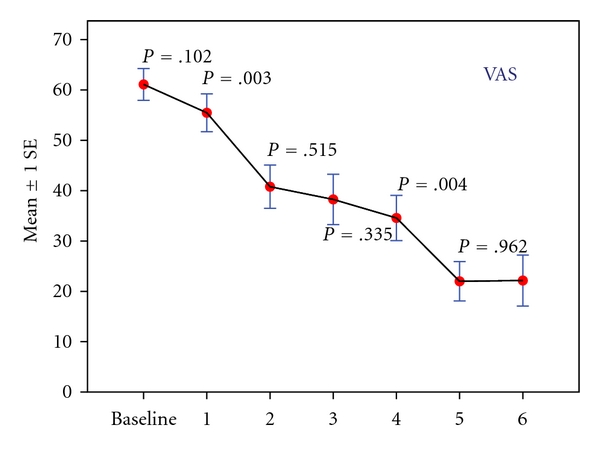
Difference between baseline and posttreatment VAS scores: VAS scores of all the patients, except one, decreased by more than 20 mm after the fourth treatment, thus showing a marked improvement in 95% of enrolled patients.

**Figure 3:**
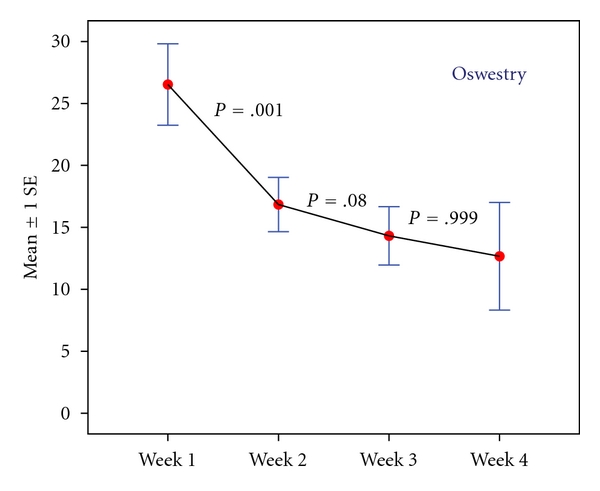
The mean ± SD baseline ODI for all participants was 26 ± 14 (range 8–64). ODI decreased throughout the entire treatment period. A significant change from baseline was notable in the third week of treatment, with a mean improvement of 14.3 ± 10 (*P* < .001).

Twenty-two patients who fulfilled the inclusion criteria were enrolled. Three of these patients were removed from the study prior to treatment initiation because they scored <40 mm on the VAS immediately before the first treatment. These dropouts were excluded from all analyses. Hence, nineteen patients (5 men and 14 women) were included in the study. Their mean age was 52.1 ± 10.8 years (median  = 56; range 27–71). The mean ± SD baseline VAS score for all participants was 61 ± 14. There were no significant changes in VAS scores between enrollment and the first treatment (55.5 ± 16.4; *P* = .10). During treatment, VAS scores decreased significantly compared to baseline by 39 ± 17 (*P* < .001). Notably, VAS scores of all the patients, except one, decreased by more than 20 mm after the fourth treatment, thus showing a marked improvement in 95% of enrolled patients.  Figure 2 shows the difference between baseline and posttreatment VAS scores. The mean ± SD baseline ODI for all participants was 26 ± 14 (range 8–64). ODI decreased throughout the entire treatment period. A significant change from baseline was notable in the third week of treatment (Figure 3), with a mean improvement of 14.3 ± 10 (*P* < .001). Lumbar flexion ROM showed a mean increase of 2.1 cm during treatment, but this change was not statistically significant.

### 4.1. Adverse Effects

No serious adverse effects were reported by any of the participants. One patient reported a mild tingling in the back, which resolved itself within 6 hours of onset.

## 5. Discussion

The current pilot study was aimed to assess the effectiveness of a new, innovative neurostimulation modality in the treatment of LBP.

Localized decrease in skin resistance is frequently associated with clinically active myofascial trigger points that are richly innervated by myelinated A *δ* fibers, the smallest in diameter (0.2–1.5 *μ*m) and most commonly present myelinated axons in peripheral nerves. Their extremely small size prevents their identification by any imaging modality. However, impedance mapping combined with smart algorithms allowed their identification due to their low impedance relative to the surrounding area [17]. Electrical skin impedance measurements are considered to be vulnerable to certain sources of imprecision, including instrument error resulting from the size, pressure, and duration of probe application as well as local skin conditions such as variable thickness, hydration, and intactness of the stratum corneum [17, 18]. To overcome these limitations, a new modality was developed (Nervo-Stim), using an array of miniature probes combined with an automatic screening capability based on impedance measurements over the back with simultaneous multipoint electric high-intensity neurostimulation.

The mechanism of the analgesic effect of the new modality is not clear. Nevertheless, considerable evidence suggests that with the type of neurostimulation utilized analgesia is achieved by activating extra segmental antinociceptive mechanisms and release of endogenous endorphins, serotonin, and cortisol [10, 11, 19, 20]. The pituitary has been implicated in analgesia mediated through the release of endorphins. The endorphin moiety travels either via the blood stream to inhibit cells in the substancia gelatinosa of the spinal cord or via a reverse portal system into the cerebrospinal fluid of third ventricle to produce effects on the periaqueductal grey matter [21].

Other nonpharmacological therapies for LBP, such as TENS units, have been used for 3 decades. These are applied on large surface areas delivering low-intensity electrical stimulation to the underlying muscle nerves (type I fibers) designed to block the pain signal (gate mechanism) to the brain with a reported effectiveness of 45% rather than 36% in placebo treatments [9]. The American Academy of Neurology has advised against the use of TENS for the treatment of chronic LBP, stating that the strongest evidence indicates that it is ineffective for this condition [22].

Percutaneous electrical nerve stimulation combines the advantages of transcutaneous electrical nerve stimulation (i.e., peripheral dermatomal-based electrical nerve stimulation) and electroacupuncture (i.e., electrical stimulation at specific acupoints via percutaneously placed needles). The main advantage of PENS over TENS is that it bypasses local skin resistance and delivers electrical stimuli in close proximity to the nerve endings located in soft tissue, muscle, or periosteum with 91% of the patients reporting that PENS was more effective than TENS in decreasing their LBP [6, 7]. 

The novel modality described herein was highly effective in producing acute analgesia in this LBP population with 95% of patients reporting a significant decrease in VAS scores after four treatment sessions. More importantly, the patients began to report more sustained beneficial effects on their level of pain and physical activity after 3 to 4 Nervo-Stim treatments (Figures 2 and 3). This may suggest that Nervo-Stim produces a cumulative analgesic effect over the course of a 3-week treatment period. The main advantages of the new device are the capacity to automatically identify LBP trigger point and to stimulate them without using needles and with no previous knowledge of their location. Thus, screening and stimulation are performed in a single 25-minute session in a “user-friendly fashion.”

The major limitations of the study design are the small sample size, the absence of a control group, and that control group without treatment may improve low back pain as time passes. For these reasons, this study should be regarded as a pilot study only. A larger, randomized, controlled trial is needed for confirmation of these preliminary results.

## 6. Conclusion

The results of the current pilot study are encouraging. The mean VAS scores of participants who received Nervo-Stim treatment showed a clinically significant reduction in back pain in 95% of patients after four treatment sessions. The decrease in pain and perceived disability, combined with the improvement in ROM, support future investigations to determine the relative effectiveness of varying frequencies and durations of electrical stimulation using this novel modality and RCT longitudinal studies in the treatment of LBP.

##  Authors' Contributions 

All authors provided concept/idea/research design, data analysis, and consultation (including review of paper before submission). Dr. Gorenberg, Dr. Shieff, and Dr. Eizenberg provided writing.

##  Conflict of Interests 

This paper was supported by a grant from Nervomatrix, Israel. Dr. Gorenberg and Dr. Eizenberg are stockholders in Nervomatrix.

## Figures and Tables

**Table 1:** Eligibility criteria.

Inclusion criteria	Exclusion criteria
(i) Age 18 years to 70 years	(i) Sciatica
(ii) Nonspecific low back pain that persisted at least 1 month and no longer than 12 months before the study.	(ii) Diagnosed spinal stenosis
(iii) Patients must have a baseline score ≥40 mm on the VAS pain scale.	(iii) Back pain potentially attributable to specific underlying diseases or conditions (e.g., pregnancy, metastatic cancer, spondylolisthesis, fractured bones, dislocated joints, disc eruption).
(iv) If taking analgesics, patients must agree to maintain a steady regimen for the duration of the study.	(iv) Unstable medical or severe psychiatric conditions or dementia
(v) *Able to provide written and verbal informed consent. *	(v) Previous back surgery
	(vi) Physically unable to undergo treatment (vii) Patients receiving worker's compensation or those involved in litigation (viii) History of pacemaker, implantable devices, or of cardiac arrhythmias (ix) Allergy or intolerance to adhesive materials (x) Clinical evidence of cardiovascular, pulmonary, renal, hepatic, neurological, hematological, or endocrine abnormalities.

(European guidelines for the management of chronic nonspecific low back pain, European Commission COST B13 Management Committee; 2004).
